# The Influence of Doping on the Optoelectronic Properties of PbS Colloidal Quantum Dot Solids

**DOI:** 10.1038/srep18735

**Published:** 2016-01-08

**Authors:** P. Papagiorgis, A. Stavrinadis, A. Othonos, G. Konstantatos, G. Itskos

**Affiliations:** 1 Department of Physics, Experimental Condensed Matter Physics Laboratory, University of Cyprus, Nicosia, 1678, Cyprus; 2 ICFO - Institut de Ciencies Fotoniques, The Barcelona Institute of Science and Technology, Castelldefels, 08860, Barcelona, Spain; 3 Department of Physics, Laboratory of Ultrafast Science, University of Cyprus, Nicosia, 1678, Cyprus; 4 ICREA-Institució Catalana de Recerca i Estudis Avançats, Passeig Lluís Companys, 23, 08010 Barcelona, Spain

## Abstract

We report on an extensive spectroscopic investigation of the impact of substitutional doping on the optoelectronic properties of PbS colloidal quantum dot (CQD) solids. N-doping is provided by Bi incorporation during CQD synthesis as well as post-synthetically via cation exchange reactions. The spectroscopic data indicate a systematic quenching of the excitonic absorption and luminescence and the appearance of two dopant-induced contributions at lower energies to the CQD free exciton. Temperature-dependent photoluminescence indicates the presence of temperature-activated detrapping and trapping processes of photoexcitations for the films doped during and after synthesis, respectively. The data are consistent with a preferential incorporation of the dopants at the QDs surface in the case of the cation-exchange treated films versus a more uniform doping profile in the case of in-situ Bi incorporation during synthesis. Time-resolved experiments indicate the presence of fast dopant- and excitation-dependent recombination channels attributed to Auger recombination of negatively charged excitons, formed due to excess of dopant electrons. The data indicate that apart from dopant compensation and filling of dopant induced trap states, a fraction of the Bi ionized electrons feeds the QD core states resulting in n-doping of the semiconductor, confirming reported work on devices based on such doped CQD material.

Progress in electronics relied heavily on the succesful implementation of intentional electronic doping to efficiently control the carrier concentration and modulate the electrical properties of semiconductors^1^. Electronics have been equally benefitted from recent research enabling the shrinkage in the device dimensions to the nanoscale resulting in improved performance and new functionalities^2^. In particular the discovery of quantum-size effects in nanometer-sized crystals^3^,^4^ triggered an enormous effort in the field of quantum dots (QDs). Breakthroughs in colloidal chemistry allowed colloidal quantum dots (CQDs) to be prepared via relatively simple and cost-efficient solution processed methods and be implemented as building blocks in novel electronic, optoelectronic and electrochemical devices^5^,^6^,^7^,^8^. Further progress towards a CQD-based electronics technology is dependent on the development of reliable methods of electronic-functionalization of the CQDs in the solid state. In complete analogy to conventional electronics, doping appears as a natural pathway, however doping of CQDs remains a widely unexplored and at the same time challenging task. Initial attempts were hindered by the rejection of intrinsic impurities by the host lattice, so part of the community effort has been directed in remote doping via charge injection into the QDs from extrinsic dopants^9^,^10^,^11^ or ligand-modulated QD reduction or oxidation^12^. However the practicality of such approaches towards a universal CQD doping protocol for devices may be challenged. Progress in the chemical synthesis of doped nanocrystals^13^,^14^ allowed the implementation of various robust approaches towards the substitutional and interstitial incorporation of extrinsic impurities into InAs^15^, PbS^16^,^17^,^18^,^19^,^20^,^21^, PbSe^17^,^22^ and CdSe^23^. Based on such approaches, device concepts such as solar cells and field effect transistors have been demonstrated. Furthermore remote and intrinsic doping studies have provided evidence of successful incorporation of ionized electrons into the QD core states and information on the optoelectronic properties of the doped CQDs.

Yet more studies are needed towards the thorough understanding of the mechanisms via which dopant atoms and ionized carriers affect the recombination of excitations and influence the energy level landscape of doped CQDs. Optical spectroscopy is a suitable non-destructive diagnostic tool that can provide insight into such fundamental questions. Motivated by a recent robust approach that demonstrated n-type PbS CQDs by substitutional aliovalent Bi atoms^21^, we report on a thorough spectroscopic investigation of the optoelectronic properties of such doped CQD solids. Doping is introduced via two different methodologies: (i) *in situ* doping during colloidal synthesis of the quantum dot material, (ii) post-synthetic doping via intented cation exchange (CX) reactions. Using the two methods, a series of samples is produced in which Bi doping in the PbS host lattice is systematically increased are coded based on the % precursor. Samples are coded based on the % precursor Bi:Pb atomic ratio and categorized as series A or series B films, referring to in-situ and post-synthetic doped samples respectively. It is noted that studies of doped material produced via in-situ doping (series A) have been reported in reference 21. Such studies have demonstrated that bismuth is efficiently incorporated as electron donor in the PbS QD lattice and the produced material can be succesfully be employed as the n-type component of a homojunction CQD solar cell. The studies also indicate the presence of Bi-induced states, approximately 0.3 eV below the LUMO level of the PbS QDs however the influence of such states on the photophysics of the CQD solids has not been studied. On the other hand no studies have been reported for post-synthetic Bi-doped CQDs such as those of the series B films reported here.

## Results and Discussion

To produce the in-situ series A material, bismuth acetate is added in the original lead precursor solution which is produced by dissolving lead oxide in oleic acid and octadecene. Along with the formation of lead oleate, bismuth aceate is also dissolved producing bismuth oleate, and the two oleate complexes co-particpate in the formation of bismuth doped PbS QDs upon addition of the sulfur precursor. Hence for series A the precursor Bi:Pb atomic ratio is determined by the original lead oxide to bismuth acetate weight ratios. The material of series B is produced via syntesis of PbS QDs that are purified and subsequently injected into a bismuth oleate solution. In this case, the weight of the original PbS QDs is used to determine the precursor Pb amount. The material is processed into films and investigated by a variety of steady-state and time-resolved optical spectroscopic techniques, employed across a wide spectral and temporal range. The optical experiments investigate the influence of dopant incorporation on the solid state electronic structure of the QDs and provide insight towards the understanding of the dopant-induced mechanisms through which they affect the recombination of photoexcitations in the doped material.

**Figure 1:**
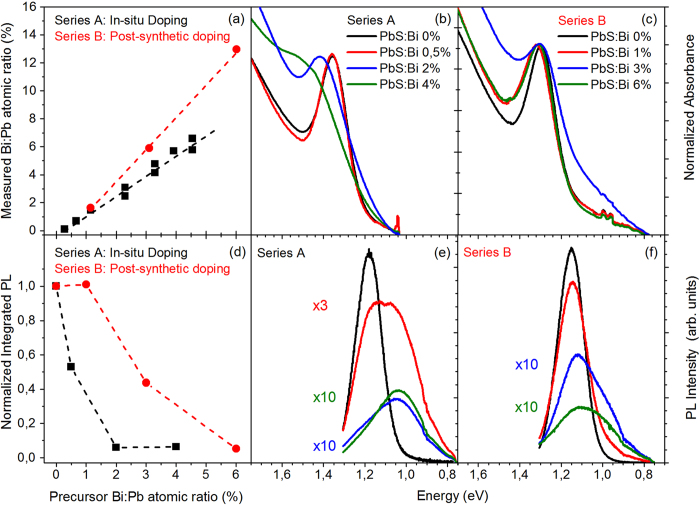
(a) Degree of Bi incorporation into PbS QDs using the two doping mechanisms provided by ICP-OES experiments. Data for doping during synthesis have been taken from^21^, **(b)** Normalized absorbance of series A (doping during synthesis) films at 300K, **(c)** Normalized absorbance of series B (post-synthetic doping) films at 300 K, **(d)** Comparative integrated PL of the films of the two series at 300 K. The integrated PL has been normalized to the respective of the undoped films, **(e)** Comparative PL spectra of series A films at 300 K, **(f)** Comparative PL spectra of series B films at 300 K.

Figure 1(a) contains the results of inductively coupled plasma optical emission spectrometry (ICP-OES) experiments on the studied material. The data confirm previous findings^21^ on the efficient incorporation of Bi in the dots during their synthesis, followed by substitution of Pb cations by Bi. In particular, for both series A and B the measured Bi:Pb ratio is higher compared to the precursor Bi:Pb ratio, i.e. 1.4 times higher and 2.2 times higher for series A and B respectively. Small deviations between the precursor and final Bi:Pb ratios, as those observed in series A, can be attributed to a favored incoproration of Bi in the PbS sructure i.e. cation exchange reactions during the QD formation and growth, or/and formation of Pb^2+^ vacancies upon Bi^3+^ incorporation in the QD structure as a charge compensation mechanism. For larger deviations such as those in series B samples, it is necessary to consider changes induced by the purification process of the doped QDs accompanying the CX reactions. The purification is performed via routine precipitation-centrifugation-redispersion cycles and results in loss of the QD material affecting the Bi and Pb amounts in the final QD product. Based on the evidence above it is reasonable to assume that in series B, Bi is mainly located in and on the surface of the QDs and that solvent dispersion of individual QDs during purification depends on individual doping with smaller loss of the QD material as more Bi is introduced.

Figure 1(b,c) contains the steady-state absorbance from the films of the two series. The absorbance has been normalized to the peak of the 1S_e_-1S_h_ excitonic QD transition. Series A exhibits a monotonic broadening, quenching and blue shift of the fundamental excitonic transition (up to ~100 meV) with dopant content. Such absorption modifications could be considered as a manifestation of the QDs conduction band charging by the ionized electrons of the dopants^9^,^15^,^21^,^24^. However the interband absorption spectral changes may also arise from other dopant-induced effects such as the introduction of above or below-gap energy states^15^,^21^, or the carrier filling of surface and trap states instead of core QD states that leads to broadening and shifting of absorbance via the Stark effect^24^.The same absorbance modifications are also observed in series B however the effect magnitude is significantly smaller compared to those in series A. i.e. 5 times smaller blue-shifts of the 1S_e_-1S_h_ optical transition. Fig. 1(e,f) contains comparative PL spectra from the studied films performed under 785 nm (~1.58 eV) quasi-resonant excitation of the 1S_h_ - 1S_e_ transition. In both series, the introduction of the dopant species results in efficient quenching of the PL intensity, red-shift of the PL peak and broadening of the PL lineshape towards the infrared. The emission quenching is quantified in Fig. 1(d) where the integrated PL for all films studied, is plotted versus the precursor Bi:Pb ratio. For series A an amount of 2% Bi is sufficient to quench the integrated PL by 95%, in close agreement with previous studies on the same material^21^. PL suppression in series B exhibits a significantly weaker dependence with Bi content. Quenching by the same fraction requires the incorporation, as measured by the ICP-OES experiments, of Bi content that is 5–8 times larger compared to series A.

Overall the data of Fig. 1 show that doping during synthesis results in stronger perturbation of the QD photophysical properties compared to post-synthetic doping using cation-exchange reactions. As discussed earlier, it is reasonable to assume that the later process may result in the anisotropic incorporation of dopant atoms on the surface versus a more uniform dopant embodiment in the PbS lattice allowed by doping during synthesis. Such a preferential inclusion of dopants should result in a larger average spatial separation and thus weaker interactions of dopants with PbS photoexcited excitons that are uniformly generated throughout the whole QD volume. The assumption is further evaluated with temperature-dependent and transient absorption and PL experiments analyzed later in the text. Another observation is that both doping methods appear to result in a more dramatic bleaching of the PL compared to absorption, in general agreement with previous studies^11^,^21^,^24^. It is noted that the comparative PL data of Fig. 1(d–f) have been normalized to the respective absorbance at the excitation wavelength so they do not include losses in emission associated with the dopant-induced absorption reduction. Luminescence compared to interband absorption is a higher ordered process that involves additional mechanisms such as the formation, energy relaxation and recombination of excitons. In particular for the PbS material system, the high dielectric constant screens the Coulombic interaction of electrons and holes lengthening the natural radiative lifetime typically to levels of hundreds of ns. So even at the level of one added charge per dot, band-edge emission in the n-doped QDs is expected to quench efficiently via the formation and Auger recombination of trion (X-) species that occurs at sub-ns scales i.e. orders of magnitude faster than radiative decay^11^. Alternative pathways of dopant-induced PL quenching may also include exciton quenching by local electric fields, carrier or exciton trapping at crystal distortion defects or trap states, and non-radiative decay via hole trapping by the electron-rich QD surface^24^.

**Figure 2:**
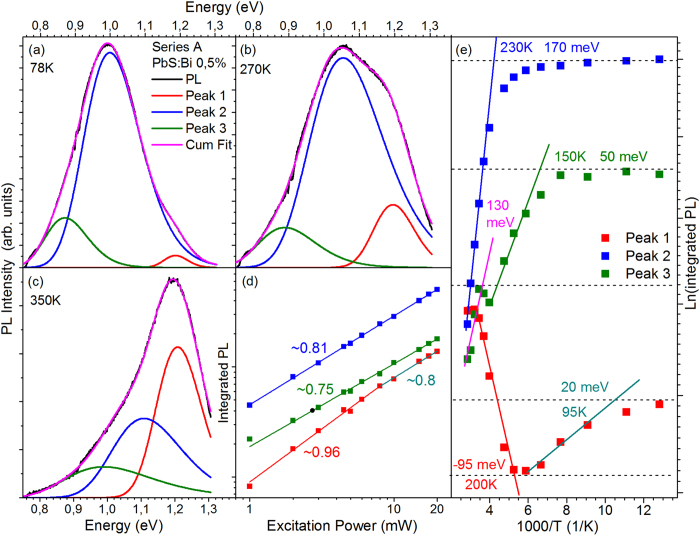
Temperature-dependent PL characteristics of the PbS:Bi 0.5% film from Series A. Triple-Gaussian fitting of the PL spectra from the film at 78 K **(a)**, 270 K **(b)**, 350 K **(c)**. **(d)** Integrated PL versus excitation power for the three PL components, **(e)** Arrhenius plot and calculated activation energies for the Gaussian components of the PL spectra.

To investigate the mechanisms of the PL bleaching and the spectral modifications of the PL lineshape induced by the Bi dopants, we performed a temperature-dependent PL study of the studied films. Samples of series A doped during the synthetic process are initially discussed. Fig. 2(a–c) contain the PL spectra at 78 K, 270 K and 350 K from the lighter-doped film of 0.5% Bi. Increase of the sample temperature appears to result in a more asymmetric PL lineshape, with a preferential quenching of the lower energy in favor of the higher energy wing of the luminescence. The anisotropic quenching of the PL spectrum induces an effective blue-shift of the PL with temperature that is superimposed to the expected PL blue shift due to the anomalous variation of the PbS gap with temperature^25^. The behavior indicates the presence of multiple contributions to the luminescence that exhibit different temperature-dependent characteristics. To analyze the different emissive components, a systematic Gaussian analysis of the PL lineshape at each sample temperature was performed. Good PL spectral fits require at least three Gaussian curves. As will be apparent later in the manuscript, the three contributions appear as general recombination channels in all studied doped films and will be referred in the manuscript from now on as peak 1,2 and 3 in descending order of energy.

In the 0.5% Bi sample, increase of the temperature from 78 K to 350 K results in an increase of the relative contribution of the peak 1 (red curve) at the expense of the lower energy peaks 2 (blue) and 3 (green). The behavior is quantified in the Arrhenius plot of Fig. 2(e). The intensity of the dominant, at low temperatures, peak 2 appears almost unaffected with temperature up to ~200 K; for higher temperatures its intensity quenches rapidly with an activation energy of ~170 meV. Peaks 1 and 3 exhibit a more complicated “s-like” dependence with temperature. Their PL intensity begins to decrease at lower temperatures of ~90 K and ~150 K, with substantially lower rates compared to peak 2. At ~200 K and 250 K, respectively peaks 1 and 3 acquire a negative slope resulting from a growth of the PL intensity with temperature. The increase is more substantial and occurs within a larger temperature range for the high energy peak 1. The cumulative behavior of the three components is consistent with a thermally-activated process that redistributes the photoexcited species responsible for the radiative decay of peak 2 mainly to the respective species of peak 1 and to a smaller degree to those of peak 3. Consistent with the model, the measured activation energy of peak 2 matches its energy separation with the higher energy peak 1 of the PL lineshape. It is noted that such a temperature-activated behavior is not observed in any of the reference undoped films of the series, which are found to exhibit a monotonic quenching of the PL integrated intensity with temperature and activation energies of 70–75 meV that are fairly typical of oleic-acid capped PbS QD solids as observed in Suppl. Fig. 1(b,c). The anomalous temperature behavior of the PL can thus be attributed to photoexcitation interactions induced by the dopant atoms. It is noted that the PL spectra of the two undoped films exhibit a slightly anisotropic lineshape at elevated temperatures, with the degree of anisotropy though considerably smaller compared to that observed in the doped QDs. Gaussian linefitting of an undoped film at 300 K, observed in Suppl. Fig. 1(d) reveals as the origin of the PL anisotropy a weak contribution due to a low energy feature at ~110 meV below the dominant Gaussian curve. In the reference sample of series A, the contribution of this lower energy feature on the integrated PL is less than 5% at 300 K within the uncertainty of the curve-fitting process; in the undoped film of series B displayed in Suppl. Fig. 1(d) such contribution is somewhat larger. The two PL components are most probably associated with the lifting of the degeneracy due to coupling of equivalent L-valleys and splitting of the lowest electronic transition in PbS QDs. Such splitted peaks have been previously observed at separation energies that match the energy separation of the two Gaussian contributions in our data i.e. ~120 meV in PbS QDs of 2.7 nm in diameter^26^ that is not too different in size from the QDs of the present study.

A question raised here is whether the low energy peaks 2 and 3 observed in the doped QD samples are related to the L-valley split-contributions of the reference samples. To further probe the nature of the three recombination channels, an excitation-dependent PL study was performed at doped and undoped films at room temperature. The results of the Gaussian fitting analysis of the data from such an experiment from the 0.5% sample along with linear regression fits are displayed in a log–log plot in Fig. 2(d). The high energy peak 1, exhibits a monomolecular behavior up to moderate excitation powers and subsequently acquires a sub-linear dependence. The other two peaks exhibit sub-linear dependencies across the whole range of laser powers studied. Similar dependencies are measured in the 2% Bi film. The excitation dependence of peak 1 exhibits close resemblance to the PL dependence of the two undoped films, presented in Supl. Fig. 1(a). The variation of the excitonic band-edge emission with photoexcitation density is characteristic of colloidal QDs, exhibiting an approximately monomolecular regime at low photoexcitation densities, followed by a sublinear dependence with slopes of ~0.7 as Auger recombination becomes the dominant recombination channel (expected Auger slope of 0.67^27^). It is noted that both of the two Gaussian-resolved spectral components of the undoped QD films exhibit identical excitation behavior leaving the PL lineshape of the undoped QDs unaffected by the excitation power. This is however not the case for the three peaks of the doped QD PL, that show different PL intensity growth with excitation affecting the cumulative PL lineshape. The different excitation dependence of peaks 2 and 3 confirm that the recombination channels are not associated with a split-band of the host PbS material but are related to transitions induced by the dopant states. On the other hand, the great similarity of the excitation PL dependence and the spectral characteristics (energy position, FWHM) of the peak 1 contribution in the doped samples with the respective characteristics of the undoped sample PL as can be observed in Suppl. Fig. 2, allow us to attribute the aforementioned channel to the radiative recombination of the PbS QD free exciton.

**Figure 3:**
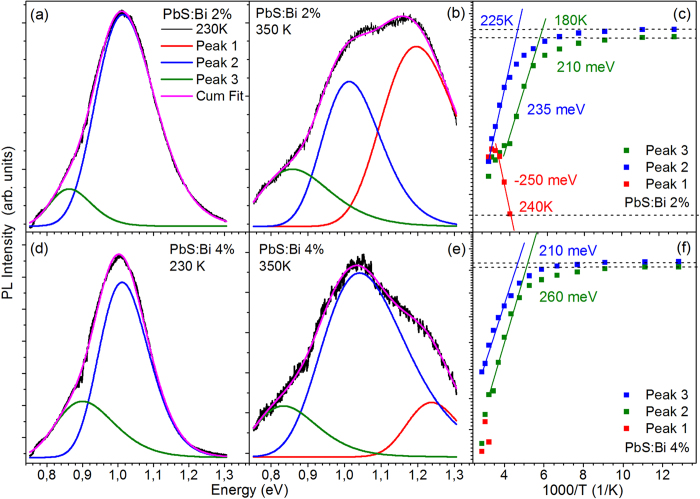
Temperature-dependent PL characteristics of the PbS:Bi 2% and 4% films from Series A. Triple-Gaussian fitting of the PL spectra from the two films at 230 K **(a),(d)** and 350 K **(b), (e)**. (**e**) Arrhenius plot and calculated activation energies for the Gaussian components of the PL spectra for the 2% Bi film **(c)** and the 4% Bi film **(f)**.

The PL temperature-dependence of the more heavily doped 2% and 4% samples of series A exhibits also a temperature-activated competition of the three emission components with the following differences compared to the lightly doped film: (i) excitations from the dominant peak 2 appears to feed almost exclusively the free exciton (peak 1) population, (ii) up to ~230 K the emission is dominated by the two lower energy components with doping resulting in systematic quenching of the free exciton contribution that appears in the PL spectra at progressively higher temperatures of ~250 K and ~310 K for the 2% and 4% doped film as can observed in Fig. 3. (iii) Higher activation energies are measured with the positive values of the lower energy peaks 2 and 3 matching exactly in magnitude the negative activation energy via which the intensity of peak 1 rises at high temperatures, conclusively confirming the presence of processes that redistribute the QD photoexcited species within the three emissive channels as temperature raises.

**Figure 4:**
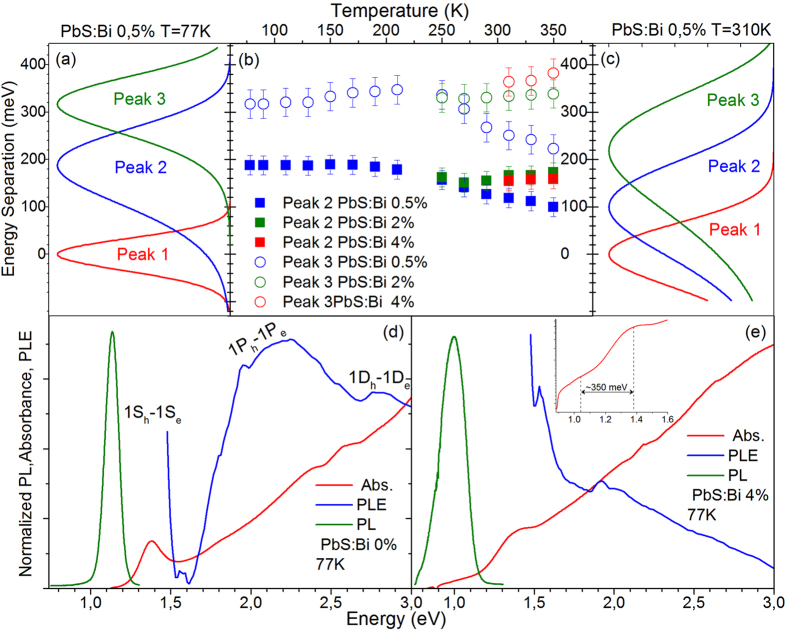
Intensity normalized, Gaussian components of the PL from the PbS:Bi 0.5% film at 78 K (a) and 310 K (c). The energy scale has been normalized to the energy position of peak 1. **(b)** Energy separation of peaks 2 and 3 from peak 1 in the 78–350 K range for all doped samples. Normalized absorbance and PLE at 78 K for the undoped film of series A **(d)** and the heavily doped film of Bi:4% **(e)**. The inset of **(e)** contains the absorbance in logarithmic scale at energies in the proximity of the 1S_e_-1S_h_ transition.

To further probe the nature of the lower energy recombination channels in the doped QD samples, we analyze their relative spectral position and its variation with temperature. Fig. 4(a,c) shows examples of the intensity normalized Gaussian PL contributions of the 0.5% Bi sample with the energy scale normalized to the high energy peak 1. A direct observation is that the lower energy PL components approach in energy the free PbS QD exciton as temperature raises from 78 to 310 K. The summary plot of Fig. 4(b) shows the relative position of peaks 2 and 3 compared to the PbS QDs exciton (peak 1) within the 78–350 K range. In the lighter doped film the linefitting analysis yields at 78 K the peaks 2 and 3 at ~190 meV and ~315 meV below the QD exciton. The energy separation with the QD exciton decreases by approximately 90 meV to ~100 meV and ~225 meV when the temperature is raised at 350 K. On the other hand for the heavier doped QD films of 2% and 4% the relative position of peaks 2 and 3 appears almost unaffected by temperature, lying at ~160 meV and ~350 meV below the band-edge emission.

Further information on the spectral behavior of the low energy recombination channels is provided by low temperature absorption and excitation PL (PLE) experiments. The results of such experiments for the reference and the heavier doped film of 4% are displayed in Fig. 4(d,e) respectively. For the undoped PbS QD film the PLE signal exhibits an initial rise as the excitation energy decreases with visible contributions from the allowed excitonic transitions 1P_e_-1P_h_ and 1D_e_-1D_h_ and potential contributions from nominally forbidden transitions between S-P and P-D quantum confined states^28^,^29^. For lower energies the signal drops rapidly as the density of QD confined states decreases below the P-states before rising again due to the 1S_h_-1S_e_ transition. No significant tailing of the absorption is observed towards the infrared and radiative recombination is Stoke-shifted by ~220 meV.

The heavily-doped sample exhibits a different behavior. The Stokes shift almost doubles at ~400 meV and the absorbance visibly extends towards low energy indicating the formation of Urbach band-tailing. Within the absorption tail a weak, broad sub-gap feature at ~0.35 eV below the 1S_h_-1S_e_ transition can be observed in the inset of Fig. 4(e). The feature spectrally coincides with (i) the energy separation of the free exciton (peak 1) with the lowest energy contribution in the doped QDs (peak 3), (ii) a redox peak appearing in the cyclic voltametry spectra of samples with very similar characteristics studied elsewhere^21^. Based on such evidence and taking into account the sublinear PL excitation, its temperature-dependent behavior and its approximately independent of dopant-content energy position, we assign peak 3 to a radiative transition from Bi-induced deep states (~0.3–0.35 eV) below the conduction band to the valence band of the PbS QDs i.e. a donor to valence band transition^30^. Figure 4(e) contains also the 77 K PLE of the 4% doped sample that exhibits a markedly different behavior compared to the respective undoped QD film spectrum. The signal appears weak at high excitation energies and monotonically increases as the excitation energy decreases, while contributions due to optical transitions between QD confined states appear to broaden and weaken compared to the PLE of the reference film. The PL spectrum of the doped QD film at 77 K is dominated by peak 2, so the PLE signal provides spectral information on the photo-excited species responsible for this dopant-induced recombination channel. Based on the behavior of the PLE data along with the temperature and excitation-dependent PL presented in Figs 2 and 3 we attribute peak 2 to the radiative recombination of excitons bound to Bi donor atoms. Impurity atoms within a host crystal act as efficient traps of free excitons converting them into bound excitons^31^. Donor-bound excitons typically dominate the low temperature emission of various n-doped bulk and nanostructured i.e. modulation-doped quantum well, semiconductor structures^30^,^31^. As the relevant emission channel can be observed over a wide range up to high temperatures where most of the Bi dopants are expected to be ionized, we can assume that such a process involves predominantly the interaction of Bi ionized donors with photoexcited PbS excitons, forming ionized donor bound excitons (D^+^X). The donor-exciton coulomb interaction lowers the exciton energy resulting in redshifted PL peak as observed in our spectra. Typical binding energies of donor bound excitons in quantum wells are in tens of meVs, compared to localization energies of 100–160 meV measured in our spectra. However it can be expected that the D^+^X binding energy in the QDs would be significantly raised due to the strong confinement, especially since small size QDs have been used in our studies. The temperature-activated detrapping processes of Figs 2 and 3 is a characteristic signature of impurity-bound excitons that thermally ionize to feed the free exciton population resulting in the observed increase of the PL emission with temperature. The interpretation is also consistent with the rather peculiar spectral shape of the low temperature PLE of Fig. 4(e) and the very weak luminescence from the doped samples under high energy excitation. Excitons bound to ionized D^+^ donors are stable when the participating holes do not possess enough kinetic energy to break away from the D^+^-electron fraction of the bound exciton complex^31^. High energy excitation can provide adequate excess of hole energy to destabilize the bound exciton species with the resulting hot carriers being susceptible to non-radiative recombination via processes such as Auger.

**Figure 5:**
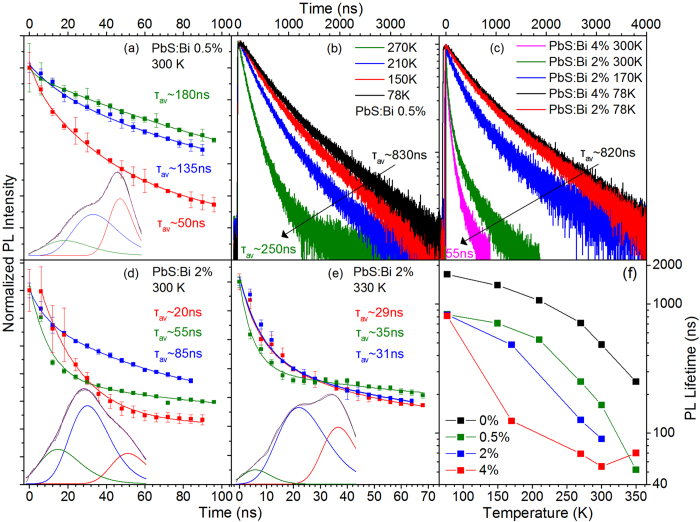
Temporal PL characteristics of the films of Series A. Time-resolved PL decays of the 0.5% Bi film at 300 K **(a)**, 2% at 300 K **(d)** and 2% at 330 K **(e)**. The decays are produced via triple Gaussian linefitting of the temporal evolution of PL with a step of 6 ns. The lower part of the figures contain examples of PL timeshots and the respective Gaussian-fitted components. **(b)** Cumulative PL decays from the 0.5% film in the 78–270 K. **(c)** Cumulative PL decays from the 2% and 4% films in the 78–300 K range. **(f )** Cumulative PL lifetimes versus temperature of the films of series A.

The effect of doping on the recombination dynamics was measured via temperature-dependent time-resolved PL experiments. The analysis of the transient decays is separated into two different temperature regimes. In the low temperature regime of ~78–270 K, recombination on all doped films is dominated by the intermediate PL peak assigned to the D^+^X channel, as observed in Figs 2 and 3. Thus TR-PL decays monitoring the peak of the luminescence, within an energy bandwidth of 20 meV, essential probe the dynamics of the bound exciton transition. The results of the study are contained in Fig. 5(b,c). All decays can be adequatly described by double-exponential fits. At 78 K, the average PL lifetime of the bound exciton appears unaffected by the doping content with values of ~830 ns, ~810 ns and ~820 ns for the 0.5%, 2% and 4% Bi doped QD solids, respectively. As temperature is raised up to 270 K the lifetime of the D^+^X species quenches to ~250 ns, ~120 ns and ~70 ns, for the 0.5%, 2% and 4% doped QD films that amounts to a lifetime reduction of ~70%, ~85% and ~91% respectively. Temperature-activated non-radiative recombination that is not related to doping, quenches the PL lifetime of the undoped reference sample by 58% for the same temperature range as can observed in Suppl. Fig. 1(f). As expected the bound exciton is susceptible to additional temperature-activated channels that quench its lifetime faster compared to that of the free exciton, such as the bound-to-free exciton conversion observed in the PL temperature-dependence of Figs 2 and 3. It appears that increase of the doping level results in a rate increase of such recombination channels.

For higher temperatures in the range of 270 to 350 K, the free exciton gains in intensity at the expense of the two dopant-recombination channels. As a result there is significant spectral overlap of the three components and TR-PL decays recorded at the PL peak essentially probe a convolution of the time dynamics of the different peaks. To differentiate the temporal behavior of the three peaks we performed a TRES experiment, the characteristics of which are clarified in the experimental section of the manucript. Analysis of the TRES experiment yields a time sequence of integrated PL spectra with a time step of 6 ns that are linefitted with triple Gaussian curves. The process is rather elaborate but ensures high spectral selectivity and individual monitoring of each of the three emissive channels. Examples of this analysis are presented in Fig. 5(a,d,e) for the 0.5% and 2% film. All decays obtained can be fitted by biexponential decays in agreement with cumulative PL decays obtained under the same experimental conditions. The analysis yields in general longer average lifetimes as lower energy transitions are probed i.e. τ_peak 1_ < τ_peak 2_ < τ_peak 3_. Average PL lifetimes for the free exciton appear to be one order of magnitude lower than the lifetimes obtained using an identical analysis of the transient data in the reference samples (peak 1 in Suppl. Fig. 1(e)). The mechanisms of this lifetime quenching is hinted by the lifetimes of the respective biexponential fits. At all temperatures the fits consistently yield an ultrafast component of the free exciton relaxation in the range of 1–5 ns and a longer time contribution of 40–60 ns. The relative weight of the first term increases with temperature resulting in a systematic quenching of the average lifetime. The timescale of the fast decay is characteristic of Auger recombination due to multi- and/or charged excitons. Multi-exciton generation is unlikely at the low excitation densities and repetition rates that the time-resolved PL experiment was performed (see experimental details section). This is confirmed by the absence of multi-exciton effects and the appearance of short PL lifetimes that would signature significant Auger recombination in the transient decays of the undoped QD films under identical experimental conditions (Supl. Fig. 1(e)). It is thus most likely that Auger results from formation of negatively charge trions which indicates the presence of excess of electrons in the QD core states. This appears as a first confirmation that a fraction of the ionized electrons of the Bi dopants are indeed populating the 1S_e_ state of the doped QDs. An interesting observation in Fig. 5(d,e) is that for the 2% Bi doped sample, an increase of the temperature by only 30 K results in rapid quenching of the PL lifetime of the bound exciton and the donor to valance band transition by 63% and 37%, respectively, at the same time increasing the free exciton lifetime by ~45%. The increase of the free exciton lifetime with temperature appears to also result in an overall increase of the cumulative PL lifetime of the heavily doped 4% sample as can be observed in the summary plot of Fig. 5(f). The lifetime lengthening of the free exciton and the simultaneous rapid quenching of the bound exciton lifetimes is most probably associated with the temperature-activated conversion of the later to the former complex in consistency with the results of the PL temperature study.

The summary plot of Fig. 5(f) contains the cumulative PL lifetimes of all films versus temperature and confirms the general trends discussed above. Recombination rates in the doped QDs appear to increase faster than the respetive rates in the undoped QDs as temperature increases, attributed to the additional channels that ionizes the bound exciton transition. At temperatures of 300  K or higher though a slow-down of the lifetime quenching is visible in the heavily doped 2% and especially on the 4% sample as a result of the redistribution of photoexcited species between the bound and free exciton channels that raises the free exciton lifetime resulting in an overall lengthening of the PL lifetime.

**Figure 6:**
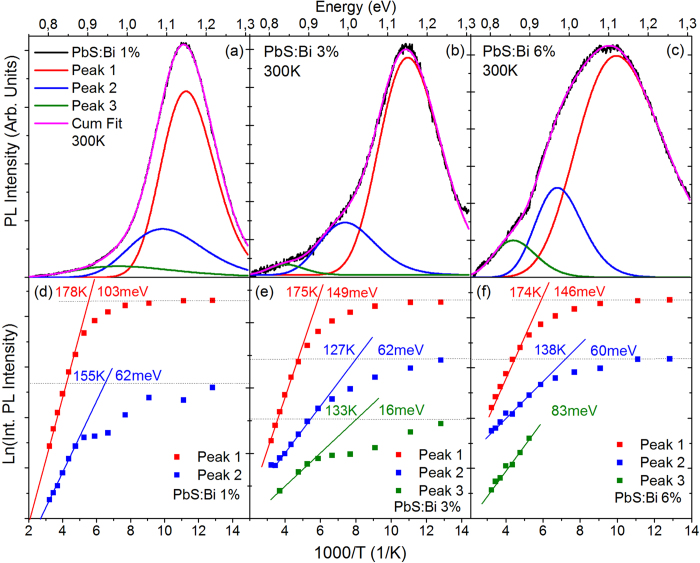
Temperature-dependent PL characteristics of the Series B films. Room temperature triple-Gaussian fitting of the PL spectra from the 1% **(a)**, 3% **(b)** and 6% **(c)** Bi film. Arrhenius plots and calculated activation energies for the Gaussian components of the PL spectra for the 1% (**d**), 3% (e) and 6% (**f** ) film.

**Figure 7:**
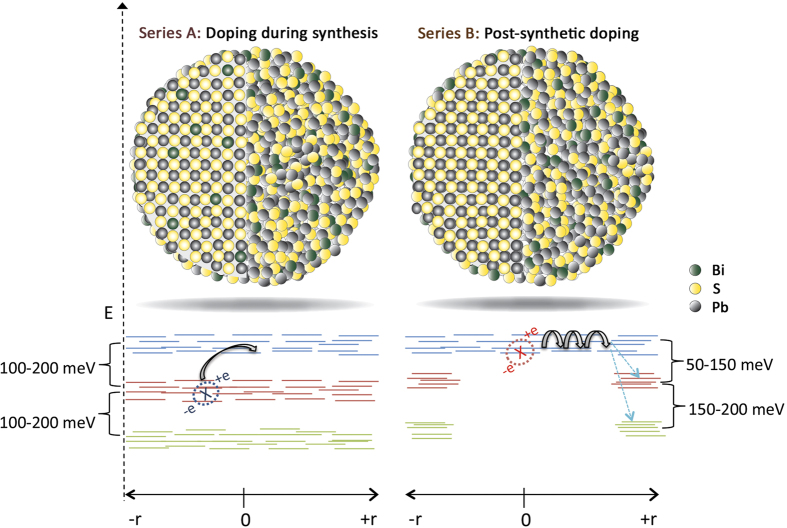
Schematic displaying the energy level landscape of the photoexcitations in the two types of doped CQDs. Temperature-dependent characteristics of the photoexcitations obtained by PL and PLE experiments are schematically shown. In films doped during synthesis a uniform distribution of dopant-induced recombination channels promotes temperature-activated detrapping processes. For films doped post-synthetically by cation exchange reactions, the preferential distribution of such states on the CQD surface denefits exciton diffusion and temperature-activated trapping processes.

The results of the temperature-dependent PL study of the series B films in which Bi doping is introduced via CX reactions, are shown in Fig. 6. Examples of the steady-state PL spectra and associated Gaussian linefitting analysis are shown for the 1%, 3% and 6% films in Fig. 6(a–c) respectively. The PL spectra require a fit by three Gaussians replicas, the spectral characteristics of which i.e. energy position, and FWHM appear to be similar with the characteristics of the respective Gaussian contributions observed in series A films. Naturally we attribute the three Gaussians to the same recombination channels with those in series A films. However the following distinct differences can be observed in series B films: (i) Free exciton (peak 1) dominates the radiative recombination during the whole temperature range probed and for all Bi dopant-levels of the films studied i.e. up to 6% Bi. (ii) The relative-contribution of the dopant-induced recombination (peaks 2 and 3) increases as temperature increases. This results from the faster temperature-activated quenching rates of peak A compared to the quenching rates of peaks 2–3 as quantified by the respective activation energies i.e. 100–150 meV for peak 1 compared to 20–80 meV for peaks 2,3. So even though the dopant-induced peaks 2 and 3 start to quench at lower temperatures of ~120–150 K compared to temperatures of ~175–200 K for peak 1, their lower quenching rate with temperature, increases overall their relative PL contribution at higher temperatures. Therefore while in films doped during synthesis, the PL data contain clear signatures of temperature-activated detrapping processes that enhance the population of the free excitons, films doped post-synthetically are characterized by temperature-activated trapping processes that convert free excitons to bound excitons or trapped excitons in Bi-induced states. The two mechanisms are schematically displayed in Fig. 7. (iii) Peak 3 attributed to the optical transition from localized Bi-induced states to the QD valence band appears quenched compared to films of series A or overall absent in the case of the lighter 1% doped film for the greatest range of probed temperatures. This suggests that post-synthetic doping either introduces a smaller density of sub-gap states or that such states are less populated compared to the case of in-situ synthetic doping. The points (i), (ii), (iii) above are consistent with our preliminary assumption that post-synthetic cation exchange doping results in a more anisotropic incorporation of the dopants on the dot surface compared to a more uniform dopant inclusion allowed by doping during material synthesis. In the former case an increase of temperature can facilitate exciton migration to the surface and enhance recombination via the surface-rich dopant states. On the other hand at lower temperatures exciton diffusion is inhibited and the larger average spatial separation of excitons with the surface Bi atoms results in their weaker interactions in favor of the free exciton recombination.

**Figure 8:**
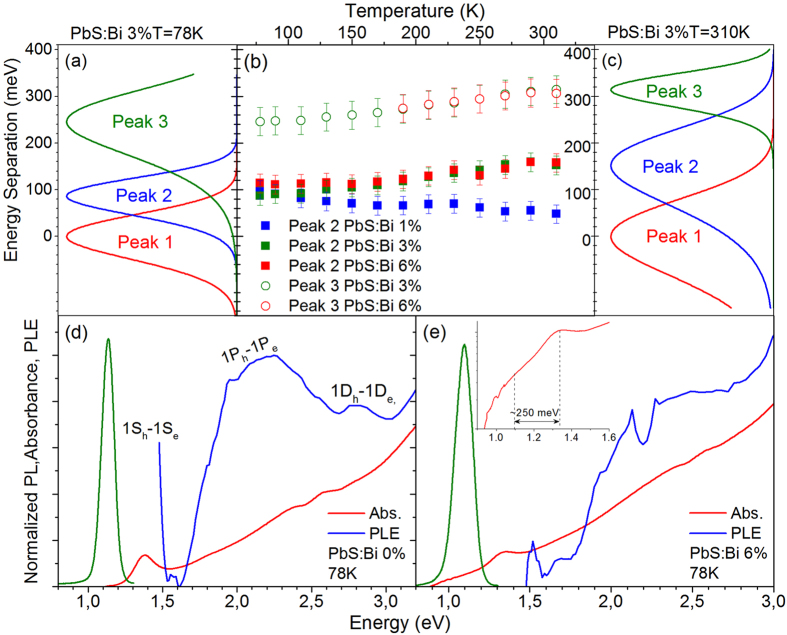
Intensity normalized, PL Gaussian peaks of the PbS:Bi 3% film at 78 K (a) and 310 K (c). The energy scale has been normalized to the energy position of peak 1. (b) Energy separation of peaks 2 and 3 from peak 1 in the 78–350 K range for samples PbS:Bi 1%, 3% and 6%. Normalized absorbance and PLE at 78 K for the undoped film **(d)** and the heavily doped film of Bi:6% **(e)**. The inset of **(e)** contains the absorbance in logarithmic scale at energies in the proximity of the 1S_h_-1S_e_ transition.

The energy separation of peaks 2 and 3 relative to the free QD exciton for the films of series B and their variation with temperature is plotted in the summary of Fig. 8(b). Peak 2 appears within a range of 50–150 meV below the band-edge emission compared to a separation of 100–200 meV in the films of series A, while peak 3 is found at 250–310 meV below the exciton transition compared with 230–380 meV in series A samples. On the average the two dopant-induced recombination channels in CX-doped films appear in closer proximity to the free exciton transition by ~50 meV. Interestingly the separation of the bound exciton (peak 2) in respect to the QD free exciton in the lighter doped film of the series (1%) decreases as temperature is raised, following the same trend with the lighter doped sample (0.5%) of series A, in Fig. 4(b). In particular the energy separation with the QD exciton decreases by approximately 50 meV from ~100 meV at 78 K to ~50 meV at 350 K in the 1% Bi film compared to a reduction by 90 meV observed in the 0.5% Bi film of the series A. The appearance of the same trend in both samples as well as the magnitude of the effect that is significant larger than the uncertainty introduced by the Gaussian linefitting analysis indicate that the temperature-dependent reduction of the bound exciton localization energy is not a spurious but rather systematic observation. On the other hand for the heavier doped QD films of 2% and 4% peaks 2 and 3 appear almost unaffected by the temperature, lying at ~160 meV and ~350 meV below the QD free exciton (values represented as dotted lines in the Fig. 4(b)).

Further insight into the recombination mechanisms can be obtained by low temperature absorption and PLE experiments. The respective experiments for an undoped (Fig. 8(d)) and the most-heavily doped film of the series are shown in Fig. 8(d,e), respectively. As in the case of the heavy doped QD films of series A, band-tailing below the 1S_e_-1S_h_ transition is observed in the 6% Bi film. However the Stokes shift compared to the undoped QD film increases only by ~20% instead of doubling and the sub-gap absorbance feature observed, appears weakened and shallower i.e. at energies of ~0.25 eV instead of ~0.35 eV below the 1S_e_-1S_h_ transition. In our previous discussion, the feature was associated with deep Bi levels, and peak 3 was assigned to radiative transitions from such levels to the QD valence band. Peak 3 at 77 K appears in series B films at ~250 meV below the band-edge emission compared to ~320 meV in series A films i.e. a difference of ~70 meV that appears consistent with the shallower by ~100 meV states in series B films and confirms the feature interpretation. On the other hand the 77 K PLE spectra of the heavily doped samples of the two series exhibit a markedly different behavior i.e Fig. 8(e) versus Fig. 4(e). Contrary to the rather peculiar shaped PLE of the 4% Bi film of series A and its mismatch with the undoped QD signal, the PLE of the series B film exhibits a spectral dependence that qualitatively matches both the film absorbance as well as the respective PLE of the reference film, even though optical transitions between QD confined states appear somewhat broadened and weakened. The PLE spectrum resemblance of undoped and CX-doped film, confirms that the predominant photoexcited species probed in both cases is of the same nature i.e. the free exciton radiative recombination.

**Figure 9:**
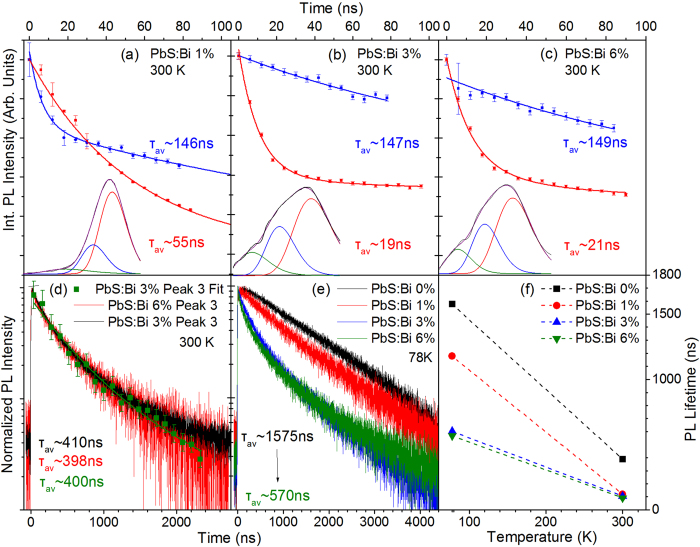
Temporal PL characteristics of the series B films. Time-resolved PL decays of the 1% **(a)**, 3% **(b)** and 6% **(c)** Bi films at 300 K. The decays monitor peaks 1 (red) and 2 (blue) produced via three Gaussian linefitting of the temporal evolution of PL with a step of 6 ns. The lower part of the figures contain examples of PL timeshots with the respective Gaussian-fitted components. **(d)** PL decays of the lower energy feature (peak 3) produced using the TRES procedure (green) along with conventional TR-PL decays monitoring the lower energy wing of peak 3 that exhibits no spectral overlap with peaks 1 and 2. The identical decays of the two methods confirm the validity of the TRES analysis. **(e)** Cumulative PL decays from all the films at 78 K. **(f)** Cumulative PL lifetimes versus temperature.

Information on the transient characteristics of the three emissive channels of series B films is provided in Fig. 9. At room temperature, decays are provided by the analysis of the TRES experiment discussed previously. All transients are described by bi-exponential fits, as in series A films. The dopand-induced peaks 2 and 3 exhibit average PL lifetimes of ~150 ns and ~400 ns that are largely unaffected by doping in the 0–6% Bi range studied. This contrasts with the behavior of films doped during synthesis where recombination rates of peaks 2 and 3 were found to strongly increase with Bi doping, as observed in Fig. 5. As a consequence the dopant-induced recombination channels in series B exhibit overall significantly slower dynamics compared to the respective channel in series A films. For example the average PL lifetime of the lower energy transition 3 appears lenghtened by a factor of ~2, ~8 and ~10 compared to the respective lifetimes on the 0.5%, 2% and 4% Bi samples of series A. The significantly smaller recombination rate is consistent with the smaller relative contribution of the Gausssian peaks 2 and 3 in the steady-state PL lineshape and appears to support our original assumption based on which post-synthetic doping introduces a smaller and/or more localized and less populated density of sub-gap states. On the other hand, the free exciton appears to be affected similarly by the two doping methods, showing a comparable lifetime reduction by a fraction of ~2.5–3 as Bi content increases from 0.5 to 4% and 1% to 6% in the series A and B films, respectively. In both cases the lifetime quenching with doping results from a higher relative contribution of a fast channel of 1–5 ns attributed to Auger recombination of negatively charge trions. As discussed previously the presence of such species can be taken as a confirmation of the presence of dopant-ionized electrons in the QD core states. Increase of temperature results in the quenching of the cumulative PL lifetime. In the low doped film of 1%, the cumulative lifetime quenching with temperature occurs at a rate similar to that observed in series A films i.e. PL transients become faster by a factor of 9 when temperature increases from 78 to 300 K. In the more heavily doped samples of 3% and 6% Bi, the cumulative PL lifetime quenches with temperature with a significantly smaller factor of ~5 due to the higher relative emission contribution of the significantly slower dopant-recombination channels 2 and 3.

**Figure 10:**
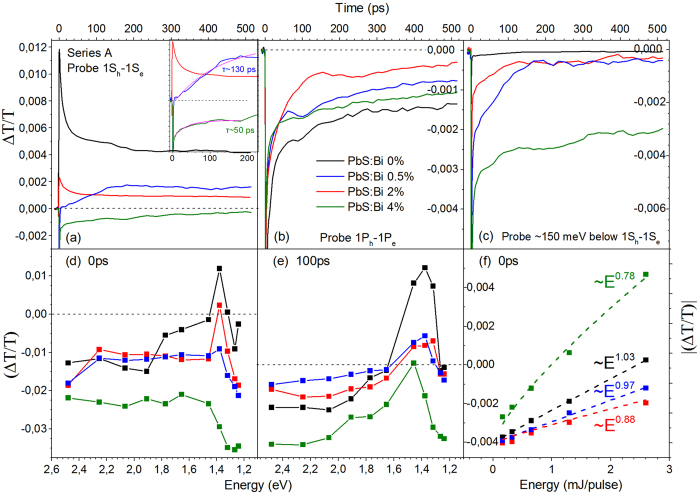
Differential transmission transients from series A films when probing the 1S_h_-1S_e_ (a), 1P_h_-1P_e_ (b) and sub-gap states ~150 meV below 1S_h_-1S_e_ (c) at 300 K. Differential transmission signals at pump-probe delay times of 0 (**d**) and 100 ps (**e**) for all measured probe energies. Absolute differential transmission at t =  0 versus pump energy along with allometric curve fits.

Ultrafast pump-probe transmission experiments with sub-ps (~100 fs) temporal resolution have complimented the PL transient study, allowing probe of the early time dynamics of the photoexcitations in the two samples series. Fig. 10 contains the results of the pump-probe study from series A films. The decays of undoped and doped films share the following common spectral and temporal characteristics: (1) Negative differential transmission signals below the 1S_h_-1S_e_ QD transition as indicated by Fig. 10(d,e) that contain the transient signals at pump-probe delay times of 0 and 100 ps, respectively. The negative signal is likely a superposition of photoinduced absorption processes of electrons, holes and excitons to higher lying energy states of PbS QDs^32^. (2) An increase of the differential transmission towards positive values in the spectral vicinity of the 1S_h_-1S_e,_ transition indicative of state filling of the S-quantized states^32^,^33^. (3) A rise signal time in the ps timescale, considerably longer than the excitation pulse width (~100 fs), characteristic of a relatively slow photoexcitation cooling time to the probed states^34^. The appearance of comparable rise times in all films suggests that the Bi dopants do not substantial influence the thermalization of the QD photoexcitations. (4) Bleaching signals are described by triple exponentials, while the majority of absorption transients by double exponentials. Both types of decays contain an ultrafast component τ_1_ of few to tens of ps and a decay τ_2_ of hundreds of ps. The bleaching signals contain in addition contributions from a longer lifetime τ_3_ at tens to hundreds of ns, beyond the temporal resolution of our setup (~500 ps) that can be confidently attributed to the recombination of singlet excitons^32^,^33^. The decay τ_1_ and to a smaller degree τ_2_ appear sensitive to the pump excitation energy, with higher pump energies resulting in faster transients as observed in Suppl. Fig. 3(a,b) that contain the bleaching and absorption signal for probes resonant to 1S_h_-1S_e,_ for the PbS:Bi 0% and PbS:Bi 4% films, respectively. The strong power dependence and the temporal characteristics of τ_1_ are highly suggestive of Auger recombination of charged excitons and biexcitons. The interpretation agrees with previous pump-probe^32^ and four-wave mixing spectroscopy studies^35^ of PbS QDs. Interestingly the transient dynamics of the heavily doped film of 4% Bi exhibit a stronger dependence with pump energy compared to the undoped film dynamics (Suppl. Fig. 3) in agreement with the results of the steady-state and transient PL transient study in which doped QD films exhibit reduced PL intensities and a larger relative weight of an ultrafast decay attributed to Auger of charged excitons. Furthermore while the decay of the reference undoped film is dominated by first-order processes as indicated by the approximately linear dependence of the differential transmission peak signal (Fig. 10c) with excitation energy (Fig. 10c), in the doped QD films the transients progressively acquire a non-linear allometric dependence typical of Auger recombination. The contribution of such non-radiative channel appears as the main cause of the efficient depletion of the QD core states resulting in a progressive reduction of the bleaching signal with Bi doping as can be observed in Fig. 10a. Probing of higher QD excited states such as 1P_h_-1P_e_ observed in Fig. 10b, results on the other hand on a progressive weakening of the photoinduced absorption signal that could be a result of a competition with a weak positive state filling signal in the doped QDs, as ionized electrons partially populate higher QD quantized states. It is evident that sub-gap states also exist and contribute to photoinduced absorption the signal of which increases as higher Bi content is incorporated in the PbS QD lattice, as seen in Fig. 10c. The energy separation of the sub-gap states probed from 1S_h_-1S_e_ appear to coincide with the energy difference of the free and D^+^X exciton PL recombination lines. Unfortunatelly the spectral capabilities of our setup do not allow probing of deeper levels in the gap of the doped QDs. A final striking observation is the appearance of growth transients, predominanlty visible in the doped QD films when probing QD excitonic transitions. The inset of Fig. 10a provides evidence of such growth signals obtained in the heavier doped films when probing the 1S_h_-1S_e_ transition with typical rise times of 50 to 150 ps. The rise signals are slow and clearly distinguishable from the ultrafast pump photoexcitation and result in a gradual filling of the 1S_h_-1S_e_ QD ground state. A plausible explanation consistent with the temperature-activation detrapping process of the PL experiments i.e. Fig. 7, is that the signal originates from donor-bound excitons that ionize at room temperature contributing to the population of free excitons and the bleaching of the 1S_h_-1S_e_ transition.

**Figure 11:**
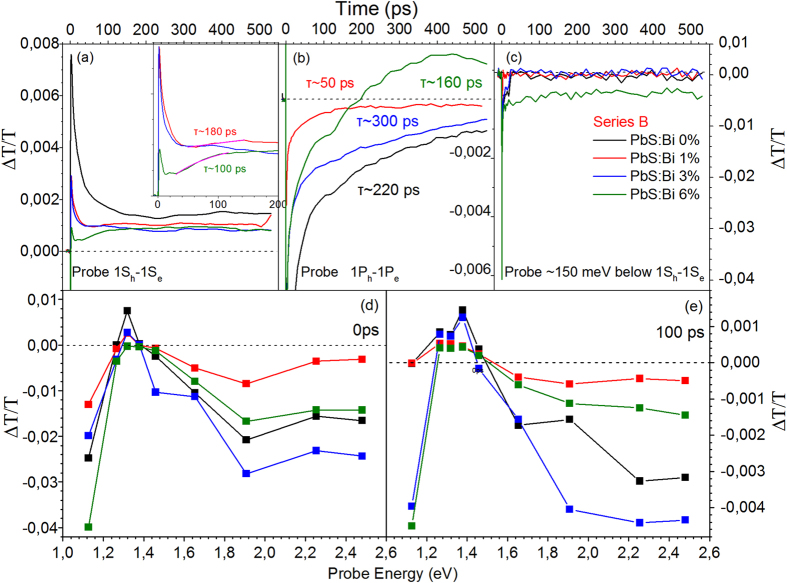
Differential transmission transients from series B films when probing the 1S_h_-1S_e_ (a), 1P_h_-1P_e_ (b) and sub-gap states ~150 meV below 1S_h_-1S_e_ (c) at 300 K. Differential transmission signals at pump-probe delay times of 0 **(d)** and 100 ps **(e)** for all measured probe energies.

The differential transmission of cation-exchange doped QD samples exhibits similarities with the pump-probe data of series A films. Increase of the Bi content results as well, in a systematic quenching of the bleaching signal when probing the fundamental 1S exciton (Fig. 11a). However the overall reduction is smaller compared to series A films and bleaching i.e. positive signal, is continusuly observed for all doping levels up to 6% in contrast to the former where photoinduced absorption prevails for Bi doping higher than 2%. Growth signals with comparable temporal characteristics to those of series A films also appear in the transients when probing the interband 1S_h_-1S_e_ and 1P_h_-1P_e_ transitions. A characteristic example is included in Fig. 11b whereby a large negative signal evolves into a substantial bleacing of the 1P exciton. In firmness with the arguments and interpretation previously given for series A films, the slow rise signals can be attributed to detrapped carriers-excitons from shallow levels induced by the Bi dopants. However the overall sub-gap absorption due to such levels is reduced in series B films, with significant signal contribution observed only for the heavily doped 6% Bi film as observed in Fig. 11c. The observation is consistent with the aforementioned PL and PLE findings that indicate a smaller density of sub-gap states induced by the post-synthetic versus the in-situ growth doping method. The contrast of Fig. 10(d–e) with 11(d)–(e) indicate an overall weaker influence on the spectral and temporal characteristics of the transients induced by doping in QD material doped using the CX reactions subsequent to synthesis.

In summary a comprehensive spectroscopic investigation of Bi (n)-doped PbS QD films is reported. Both *in situ* replacement of Pb with Bi during synthesis and post-synthetic cation exchange (CX) methods were employed to dope the QD material. A series of thin films was produced in which Bi doping was progressively increased. The spectroscopic data indicate a systematic quenching of the excitonic absorption and luminescence with doping accompanied by systematic spectral shifts and broadening. Two dopant-induced contributions at lower energies to the QD free excitons have been identified in the luminescence spectra of both types of doped films. Based on their spectral, temporal and temperature-dependent PL and PLE characteristics the channels are attributed to recombination mediated by the Bi dopants via a bound exciton complex and a donor to valance band transition. Temperature-dependent measurements provide sufficient evidence for the presence of temperature-activated detrapping and trapping processes for the films doped during and after synthesis, respectively. The data are consistent with a preferential incorporation of the dopants at the QDs surface in the case of the cation-exchange treated films versus a more uniform doping profile in the case of Bi incorporation duing synthesis. Time-resolved PL and transmission experiments indicate the presence of fast recombination channels the relative amplitude of which progressively increases with doping and excitation, resulting in PL intensity/lifetime quenching and the reduction or complete absence of exciton bleaching signals. Based on the temporal and excitation-dependent characteristics the channels are predominatly attributed to Auger recombination of negatively charged excitons, formed due to excess of dopant electrons. The data indicate that apart from dopant compensation and partial filling of trap and surface states a fraction of the Bi ionized electrons ends up feeding the QD core states resulting in n-doping of the semiconductor in agreement with reported data from processed devices based on identical doped material^21^. Fundamental studies as such provide valuable information and fundamental understanding on the vastly unknown mechanisms of dopant-exciton interactions in tiny crystals of colloidal QDs. Furthermore they can serve as a preliminary assessment of the impact of doping on the solid-state optoelectronic properties of QD films ahead of more elaborate characterization on processed devices such as solar cells and transistors.

## Methods

### Chemicals used

Lead (II) oxide (99.999%), hexamethyldisilathiane (TMS) (synthesis grade), 1-octadecene (technical grade 90%) and toluene (anhydrous, 99.8%) were purchased from Sigma Aldrich. Bismuth (III) acetate (99.999%, metals basis) was purchased from Alfa Aesar. Acetone and methanol were purchased from Panreac.

### Synthesis of PbS and doped PbS CQDs

Oleic acid-capped PbS QDs with 1S_h_-1S_e_ excitonic peak at 900–980 nm were synthesized with a modified version of a standard method^36^ using a standard Schlenk line as follows: 0.45 g of PbO was dissolved in 1.5 ml oleic acid and 3 ml octadecene under vacuum (0.2 × 10^−1^ mbar) overnight (16 hours) at 95 °C. Afterwards, 15 ml of octadecene were further added. Then, under Ar atmosphere, temperature was raised to 100 °C, and 0.21 ml TMS in 10 ml octadecene were injected, and the final solution was left to cool down to 36 °C. Then QDs were isolated/purified by precipitation upon the addition of excess acetone, centrifugation and re-dispersion in toluene under normal atmospheric conditions. This cleaning process was repeated twice with aceton. Bi doped PbS QD synthesis for series A was performed in the same way, with the addition of the appropriate amount of bismuth acetate in the original lead-precursor solution.

Post synthetic doping of PbS CQDs with bismuth, also referred as CX (cation exchange) method for sample series B was performed as follows: PbS CQDs dispersed in toluene (114 g/l) were transfered in a N_2_ glovebox. Using a Schlenk line, bismuth acetate, the amount of which was selected according to the weight/molar amount of the PbS to be used and the intended Bi/Pb atomic precursor ratio (e.g. 9.9  mg bismuth acetate for the 6% Bi/Pb sample), was dissolved in 2 ml oleic acid and 4 ml octadecene at 95 ^o^C under vacuum overnight. Subsequently the atmosphere of the bismuth precursor was switched from vacuum to Ar ad its temperature was lowered to 90 ^o^C, at which a mixture of 1.1 ml of toluene and 0.9 ml of the original PbS CQD/toluene solution was added by injection. Subsequently heating was removed and the flask was left to cool down. The final CQD product was cleaned similarly as above using once with aceton and once with a mixture of aceton and methanol as the non-solvents. All final QD products were dispersed in anhydrous toluene and stored in a N_2_ glovebox. We note that all samples of series B were made using the same original PbS QD batch.

### Film Deposition

To deposit the films, material solutions with a uniform concentration of 30 g/l were prepared. Films were deposited in ambient conditions on ~1 cm^2^ square quartz substrates using doctor-blading (Erichsen, Germany) or drop-casting. In the manuscript the former films are discussed; drop-casted films exhibit qualitatively similar PL characteristics however their larger thickness does not allow their characterization with transient absorption measurements. Doctor blading was performed at 75 °C and a speed of 15 mm/s for all studied films. A series of uniform films with average thickness of ~125 nm were produced, as measured using a Dektak 150 step profilometer (Veeco, USA).

### Optical Spectroscopy

Film steady-state absorbance was carried out using a Perkin Elmer Lamda1050 UV/Vis/NIR spectrophotometer. Steady-state photoluminescence (PL) was performed in a Fluorolog iHR320 Horiba Jobin Yvon spectrometer equipped with an infrared photomultiplier tube (PMT). Quasi-resonant excitation to the 1S_h_-1S_e_ QD transition was provided by a 100 mW 785 nm (~1.58 eV) Newport Laser Diode. An unfocused laser spot with a diameter of ~3 mm was employed in all the measurements to spatially average the PL across the films surface. Apart from the excitation-dependent experiments where the laser power was varied in the 1–25 mW range, a relatively low power of 5 mW was used to excite the steady-state PL in all other measurements. All PL spectra were corrected to account for the spectral response of the grating and the PMT of the spectrometer. To account for thickness variations within the films, the PL spectra where normalized to the absorbance of each film at the excitation wavelength of 785 nm. Photoluminescence excitation (PLE) was performed in the same setup with that of the steady-state PL experiments. PLE excitation was provided by the monochromatically filtered output of an Ozone-free 450 W Xenon Arc lamp, producing a square excitation spot of ~1 cm^2^ covering the whole surface of the films studied. An excitation bandwidth of 10 nm, corresponding to an energy bandwidth of 20–70 meV across the tuned excitation energy, was used in all experiments, while monitoring the emission at the respective PL peak within a ~50 meV spectral window. The large excitation and detection bandwidths were chosen at the expense of PLE spectral resolution, being a necessary compromise in order to obtain a presentable PLE signal-to-noise ratio from the weakly emitting doped QD films.

Time resolved photoluminescence (TR-PL) was measured on a NanoLog FL3 Horiba spectrofluorimeter, using a monochromator-based time correlated single photon counting (TCSPC) method in combination with the infrared PMT also employed in the steady-state PL measurements. The PL was excited by a picosecond laser diode at 785 nm (DeltaDiode-785L) with a pulse width of ~80 ps operating at 100 KHz, using a defocused laser beam (spot diameter ~2 mm). The system exhibits a time resolution of ~50 ps after reconvolution with the instrument response function. The PL decays were obtained while monitoring the QD emission peaks with a spectral bandwidth of ~20 meV. For the time integrated decays the aforementioned setup was used in the mode known as “Time Resolved Emission Spectra (TRES)”. The TRES experiment was used to acquire time-resolved PL decays across the whole PL lineshape with a spectral step of 5 nm. The TRES data were used to construct a three-dimensional graph containing data of PL intensity versus time and wavelength. The graph was sliced and time-integrated to obtain two-dimensional timeshots of the integrated PL intensity versus wavelength. In the analysis used in the manuscript the analysis yields integrated PL spectra decays with a temporal step of 6 ns. Each spectrum was linefitted with two (undoped) or three (doped) Gaussian curves. Each Gaussian was spectrally integrated to yield the temporal PL decay of each individual Gaussian component and avoid inaccuracies on the lifetime determination due to spectral overlap of different recombination channels. All steady-state and time-resolved PL experiments were performed under vacuum conditions (10^−3^ mbar). For temperature-dependent PL measurements the samples were loaded into a Janis liquid nitrogen optical cryostat (77–500 K).

Ultrafast time resolved pump-probe absorption measurements were carried out using a mode-locked Ti: Sapphire ultrafast amplifier generating 100 fs pulses at 800 nm running at a repetition rate of 1 kHz. A non-linear β-barium borate (BBO) crystal was used to frequency-double the amplifier output at 400 nm with energy of 1.3 mJ per pulse. The beam served as the pump excitation pulse. A fraction of the fundamental beam was used to generate a super continuum light for probing at different QD energy states in the 500–1100 nm range. Measurements were carried out using a typical pump-probe optical setup in a non collinear configuration where differential transmission was measured as a function of optical delay between the pump and the probe pulses. The probing wavelengths were selected using 10 nm narrow bandpass filters. Measurements were performed in an inert nitrogen atmosphere.

For ICP-OES, each sample was prepared by fully digesting QD powder in an aqueous nitric acid solution. Subsequently ICP-OES measurements was performed at the Scientific and Technological Centers of the University of Barcelona (CCiTUB) characterization facility using a Perkin Elmer Optima 3200 RL simultaneous ICP-OES spectrometer.

## Additional Information

**How to cite this article**: Papagiorgis, P. *et al.* The Influence of Doping on the Optoelectronic Properties of Colloidal Quantum Dot Solids. *Sci. Rep.* **6**, 18735; doi: 10.1038/srep18735 (2016).

## Supplementary Material

Supplementary Information
