# Supplementary material for: The Influence of Doping on the Optoelectronic Properties of PbS Colloidal Quantum Dot Solids
Source: Sci Rep. 2016 Jan 8;6:18735. doi: 10.1038/srep18735 (PMC4705463; doi:10.1038/srep18735)
Supplement: Supplementary Information [file srep18735-s1.doc]

**Supplementary Material**

**The Influence of Doping on the Optoelectronic Properties of Colloidal Quantum Dot Solids**

P. Papagiorgis^1^, A. Stavrinadis^2^, A. Othonos^3^, G. Konstantatos^2^ and G. Itskos^1,*^


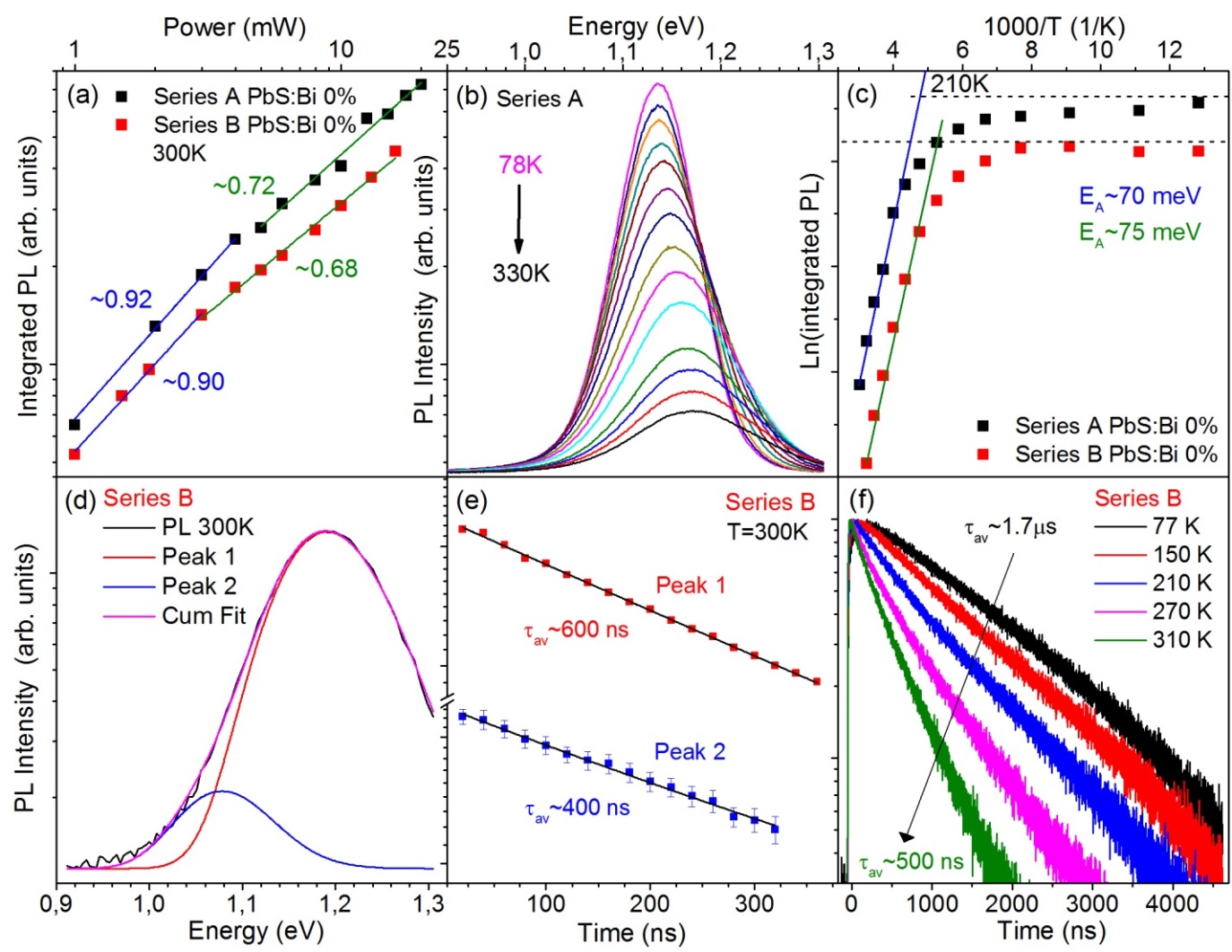


**Supplementary Figure. 1:** PL characteristics of the two reference undoped films of Series A and B. (a) Integrated PL versus excitation power. (b) PL spectra versus temperature for the series A film. The film from series B shows an almost identical behavior (c) Arrhenius plot for the two films and calculated activation energies. (d) Gaussian lineshape fitting of the series B film. The film of series A contains the same two Gaussian peaks with smaller relative weight of peak 2. (e) Time-resolved PL decay of the series B film produced via double Gaussian linefitting of the temporal evolution of PL with a step of 6 ns. (f) Cumulative PL decays from the latter film at different temperatures in the range of 77-310 K.


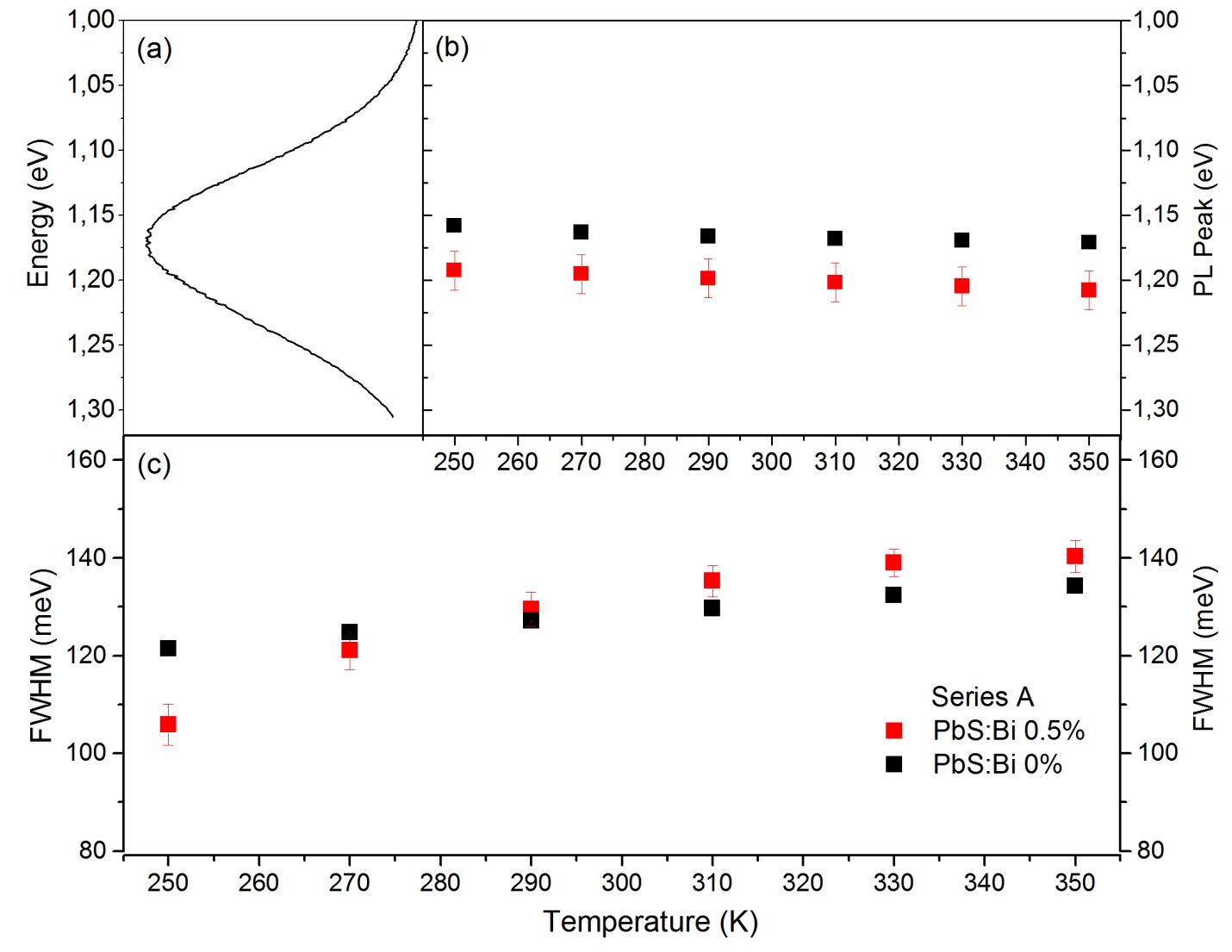


**Supplementary Figure 2: (a)** Room temperature PL spectrum of the undoped film of series A. Comparison of the PL peak and FWHM of the undoped film across the 250-350 K range with the respective characteristics of the PL Gaussian “peak 1” identified as the free exciton contribution in the 0.5% Bi film.


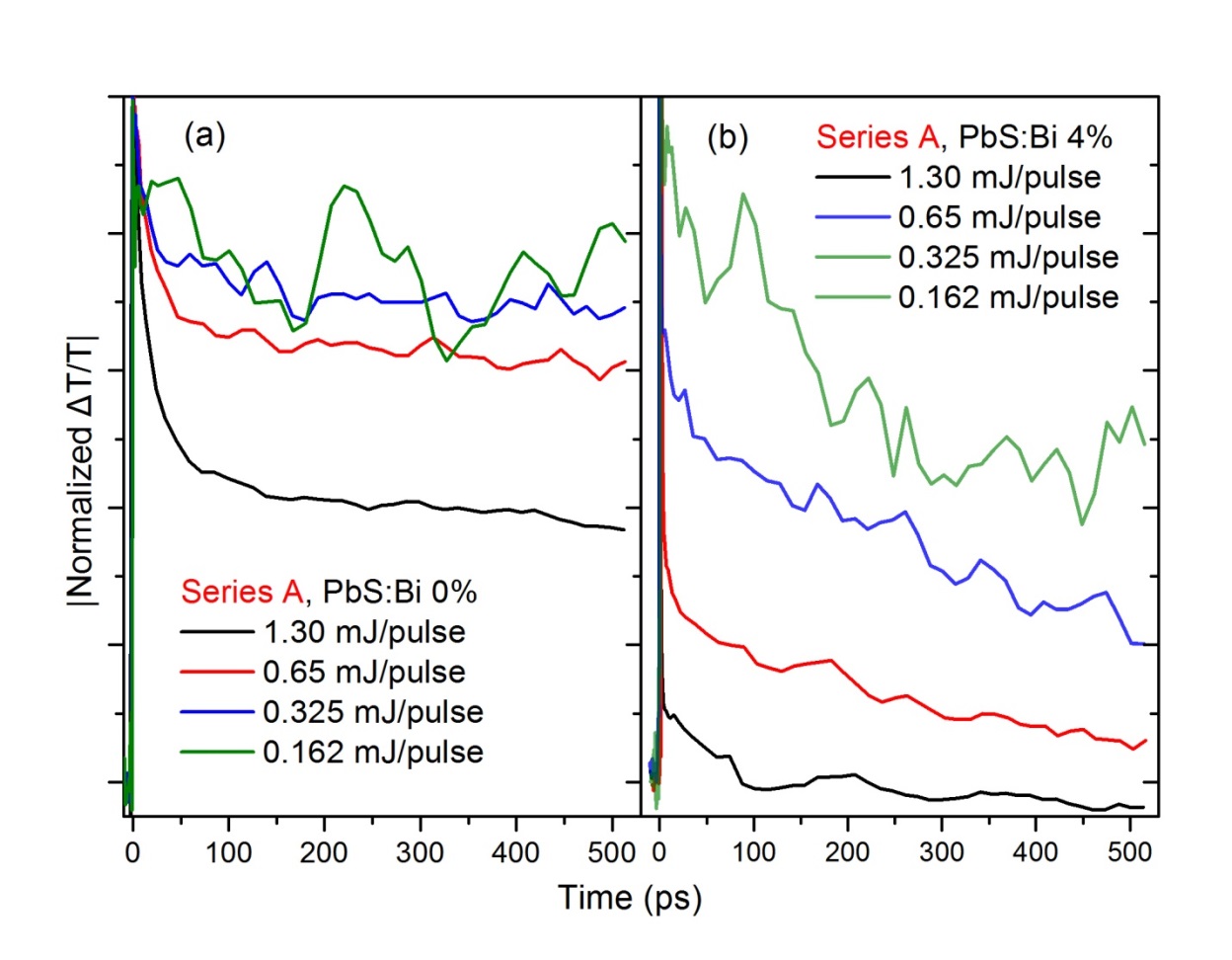


**Supplementary Figure 3:** Absolute differential transmission versus pump excitation energy when probing the 1S_h_-1S_e_ transition in the reference **(a)** and the heavily doped 4% Bi **(b)** PbS QD film produced via in-situ doping during synthesis.
